# Shallow-emerged coral may warn of deep-sea coral response to thermal stress

**DOI:** 10.1038/s41598-021-01948-2

**Published:** 2021-11-17

**Authors:** Julia W. Johnstone, Rhian G. Waller, Robert P. Stone

**Affiliations:** 1 Darling Marine Center, School of Marine Sciences, University of Maine, Walpole, ME USA; 2 Alaska Fisheries Science Center, National Marine Fisheries Service, National Oceanic and Atmospheric Association, Juneau, AK USA

**Keywords:** Marine biology, Climate-change ecology

## Abstract

In the Gulf of Alaska, commercially harvested fish species utilize habitats dominated by red tree corals (*Primnoa pacifica*) for shelter, feeding, and nurseries, but recent studies hint that environmental conditions may be interrupting the reproductive lifecycle of the corals. The North Pacific has experienced persistent and extreme thermal variability in recent years and this pattern is predicted to continue in coming decades. Recent discovery of deep-water emerged coral populations in Southeast Alaska fjords provided opportunity for detailed life-history studies and comparison to corals in managed habitats on the continental shelf. Here we show that sperm from deep colonies develops completely, but in shallow colonies, sperm development is prematurely halted, likely preventing successful production of larvae. We hypothesize that the divergence is due to differing temperature regimes presently experienced by the corals. Compared to deep populations below the thermocline, shallow populations experience much greater seasonal thermal variability and annual pulses of suspected near-lethal temperatures that appear to interrupt the production of viable gametes. The unique opportunity to comprehensively study emerged populations presently affected by thermal stress provides advance warning of the possible fate of deep corals in the Gulf of Alaska that will soon experience similar ocean conditions.

## Introduction

Red tree corals (RTC, *Primnoa pacifica* Kinoshita, 1907) are large, structure forming gorgonian corals that grow in dense thickets in some regions of the North Pacific Ocean^1,2^. This keystone species^3^ is conspicuous given its large size, tree-like morphology, and brilliant coloration, forming thickets at depths between 150 and 350 m in the eastern Gulf of Alaska^1^ (GOA). Large branching colonies up to 5 m in height and width modify small-scale ocean currents and provide extensive habitats that support high associated biodiversity^1,4^. For more than a century these corals have inadvertently been brought to the surface by fishermen, tangled in nets and lines, and have become symbolic of the rich fauna and diverse communities in Alaska’s deep marine waters.

The value of RTC thickets in Alaska to commercially important species prompted the North Pacific Fisheries Management Council (NPFMC) to designate those habitats as Essential Fish Habitat in 2000^5^. They are a common bycatch species in bottom fisheries^6,7^, have slow growth rates (1.6–2.3 cm/year), a long lifespan (commonly over 100 years)^8^, and some RTC thickets have been severely disturbed by fishing activities in the past^1^. To protect thickets from further disturbance, the NPFMC designated five small areas at two sites in the eastern GOA as Habitat Areas of Particular Concern in 2006^1,9^. Inside these closures, the use of bottom-contact fishing gear is prohibited, providing both sanctuary to the species residing there, and researchers an opportunity to study the ecology and recovery of these disturbed habitats and species.

In 2003, RTCs were discovered in the shallow waters (< 25 m) of glacial fjords in Southeast Alaska, far from their typical range offshore where important fisheries occur^10^. In habitats where temperature, irradiance, and salinity are similar to the deep sea, some usually deep-living oceanic species are able to survive at much shallower depths; a process termed deep-water emergence^10^. These populations of RTCs in the cold and dark waters typical of glacial fjords were likely established through pioneer recruitment from deep populations outside the fjords. With such deeper populations often inaccessible to researchers due to location, weather, and expense, discovery of these populations in shallow water provides a rare opportunity to access specimens for seasonal life-history studies, including reproduction^4,10,11^.

RTCs are gonochoric broadcast spawners, producing oocytes that require up to 16 months to fully develop and male gametes that develop over the course of approximately one year^11^. In both female and male colonies, multiple stages of gametes are commonly found at the same time^11^. While prior work found female colonies to be asynchronous in their release of oocytes, male colonies developed sperm in overlapping cohorts, which were spawned from September-January or March-June^11^. These characteristics indicate that RTC populations are likely sustained through sporadic recruitment events, potentially increasing their vulnerability to anthropogenic disturbances^11^. Recent comparisons of reproductive ecology among specimens from shallow-fjord sites (< 25 m) and deep-ocean sites (< 140 m) showed comparable fecundity but significant differences in oocyte size^4^. Oocytes from shallow populations were 2–3 times smaller than those from deep populations. Researchers presented two, untested, alternate explanations for the size discrepancy: (a) phenotypic plasticity, whereby shallow corals simply produce smaller oocytes in response to oceanographic and environmental conditions in the shallow fjord environment, and (b) premature arrest of gametogenesis, with the production of smaller, immature oocytes only, resulting in a reproductive dead end for the shallow populations^4^.

In this study we undertook a detailed investigation of RTC male reproductive biology, comparing spermatogenesis between shallow (< 25 m) and deep-water (> 140 m) sites in the eastern Gulf of Alaska. In addition to new ultrastructural analysis, we examined previously prepared histology slides of male colonies to compare patterns of male reproduction to previously described patterns in female colonies and explore habitat-specific effects on male gametogenesis.

## Results

**Figure 1:**
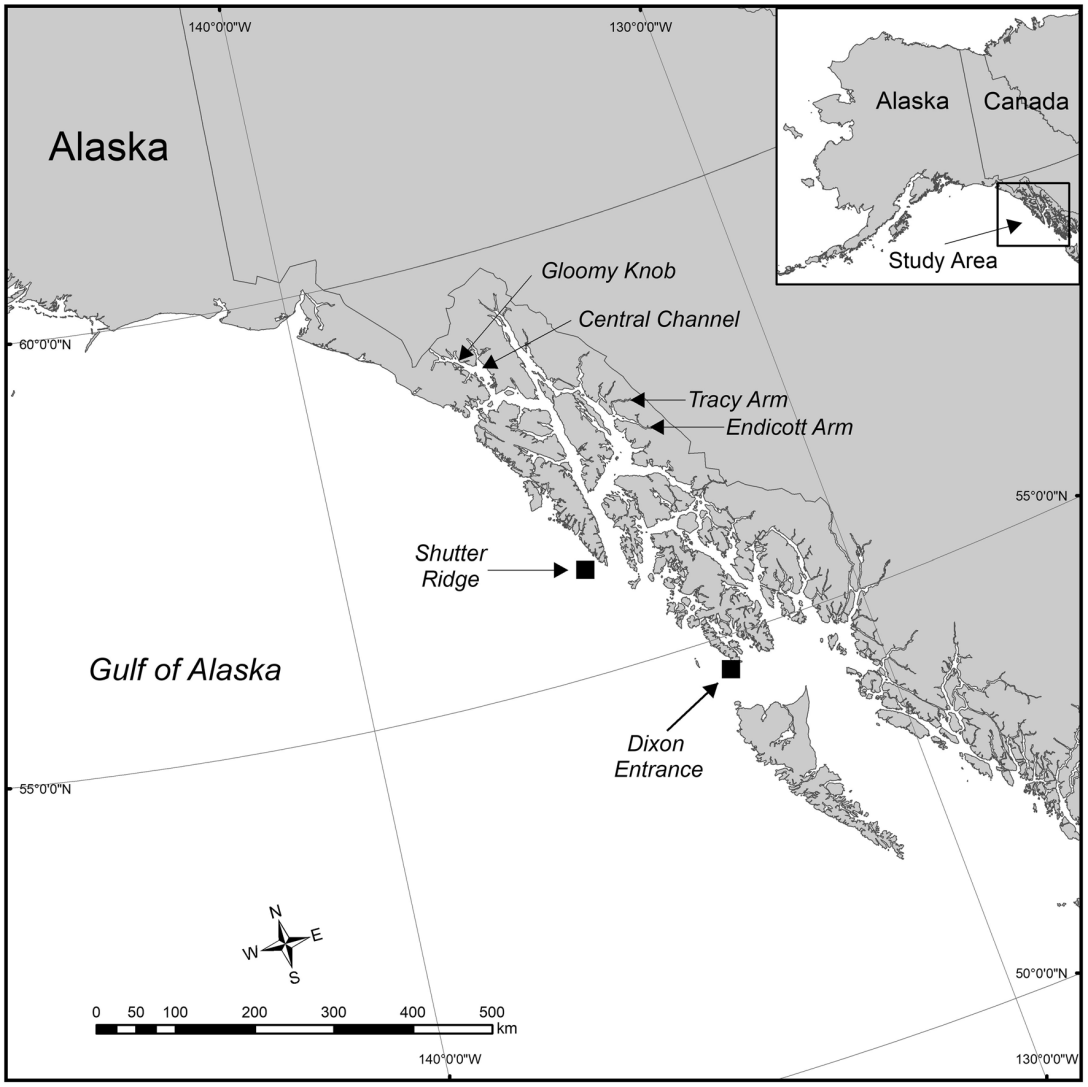
Map of the eastern Gulf of Alaska (GOA) including Southeastern Alaska where the sites studied are located. Deep ocean sites in the GOA: Shutter Ridge and Dixon Entrance. Deep fjord sites in Glacier Bay National Park and Preserve: Gloomy Knob and Central Channel. Shallow fjord sites in Holkham Bay: Tracy Arm and Endicott Arm. Map was created using ArcGIS 10.3 by Esri (https://desktop.arcgis.com/en/arcmap/).

We selected a subset of male RTC colonies from previous work^4,11^ for examination of reproductive parameters, with samples from two deep-ocean sites in the GOA, two deep-fjord sites in Glacier Bay National Park and Preserve (GBNPP), and two shallow-fjord sites in Holkham Bay (HB; Fig. 1, Supplemental Information Table S1). To determine the extent of spermatogenesis in histological samples, we measured the nuclear diameter of individual sperm cells. Due to meiotic divisions during gametogenesis, spermatocyte nuclear diameter is indicative of developmental stage, with smaller diameters representing later stages^12^.

### Deep-ocean sites

#### Gulf of Alaska: Shutter Ridge and Dixon Entrance

**Figure 2:**
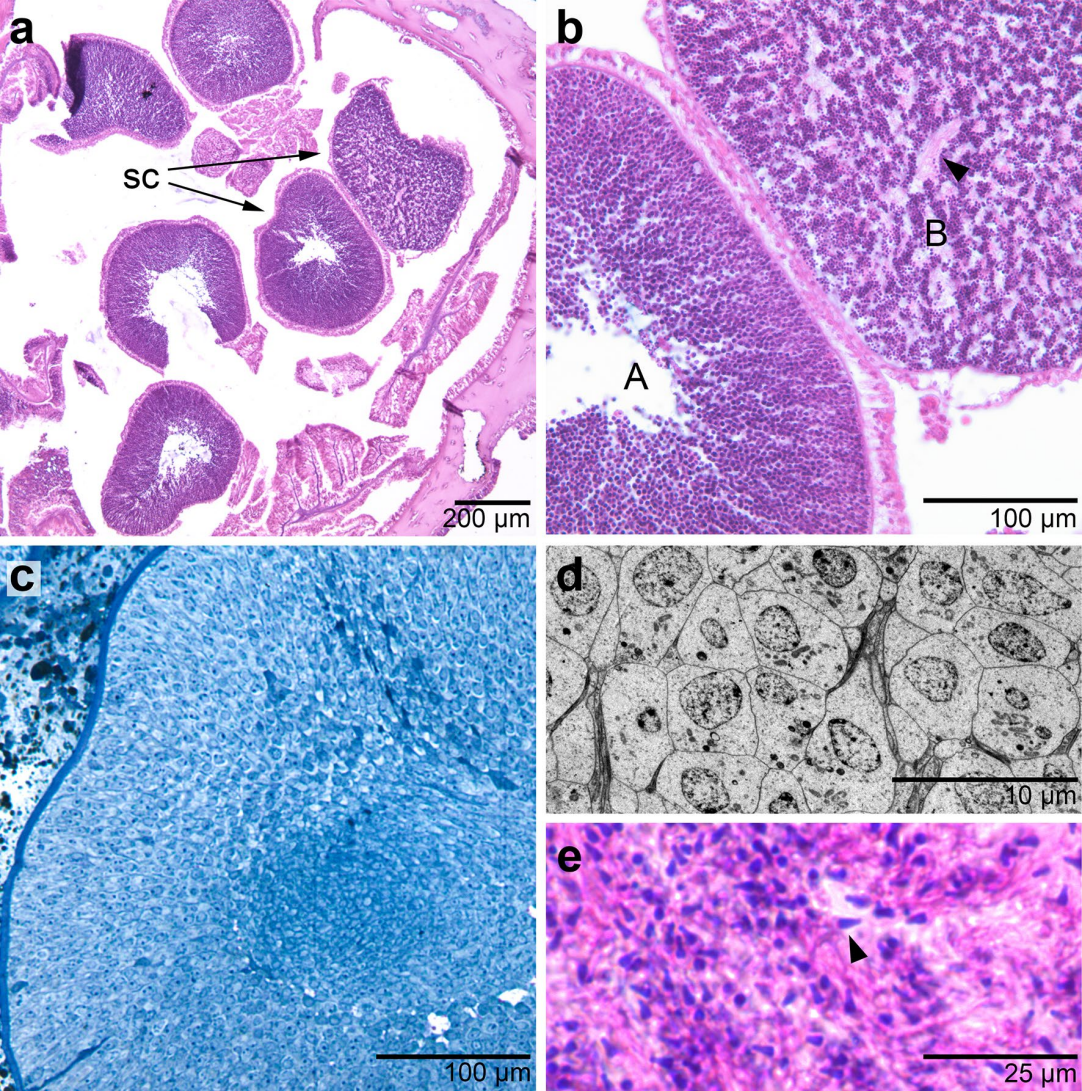
Differences in spermatogenesis between shallow and deep *P. pacifica*. (**a**) Several spermatocysts of different stages shown in histological section from a deep specimen. *sc* spermatocyst, scale bar = 200 μm. (**b**) Enlargement of (**a**): two spermatocysts from a deep specimen at an (A) intermediate stage of development and (B) a late stage of development. Note the appearance of pink bundles of sperm tails (arrowhead) in the late-stage spermatocyst not present in the intermediate stage. Scale bar = 100 μm. (**c**) Section through a representative of the latest stage spermatocyst found in any shallow water sample, embedded in resin. Scale bar = 100 μm. (**d**) Electron micrograph of the spermatocyst shown in (**c**), showing sperm cells early in their developmental process, having no ultrastructural characteristics of late-stage spermatozoa. Scale bar = 10 μm. (**e**) Light-microscope image of a spermatocyst from a deep ocean specimen containing spermatozoa with purple-stained nuclei. This latest stage of spermatogenesis is recognizable particularly by the change from a spherical to a cone-shaped nucleus (arrowhead). Scale bar = 25 μm. (**a**,**b**,**e** stained with hematoxylin and eosin; **c** stained with Richardson’s stain; **d** stained with lead citrate and uranyl acetate).

**Figure 3:**
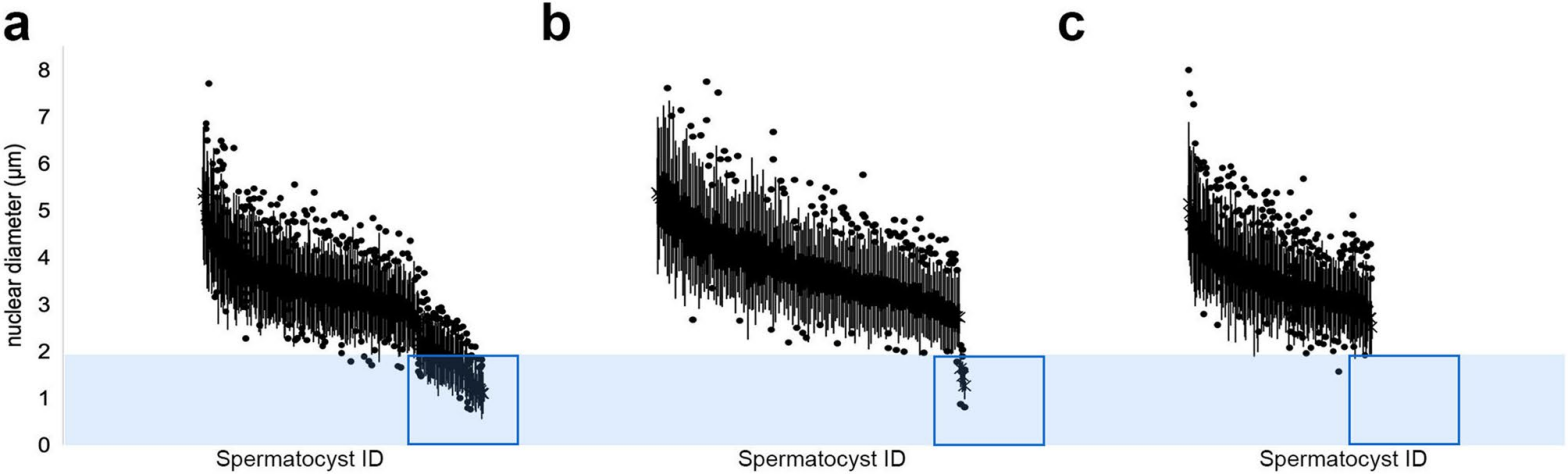
Sperm nuclear diameter in *P. pacifica* colonies from deep ocean (**a**), deep fjord (**b**), and shallow fjord (**c**) populations. Nuclear diameters of sperm cells decrease as spermatogenesis progresses^13^. Each bar represents the distribution of sperm nuclei in a single spermatocyst (see Supplemental Information Table S1 for n). The range of nuclear diameters of fully developed spermatozoa (mean > 2.25 μm) is shown by the blue rectangle. In deep water sites (**a**: GOA—Shutter Ridge and Dixon Entrance, **b**: GBNPP—Gloomy Knob and Central Channel), mature spermatozoa with such small nuclear diameters were found (boxes in **a**,**b**). By contrast, in shallow populations (**c**: HB—Tracy Arm and Endicott Arm) spermatozoa with such small nuclei were absent (box in **c**), indicating that spermatogenesis was prematurely arrested.

Samples collected from deep-ocean sites, Shutter Ridge and Dixon Entrance, in the GOA contained a full complement of the four sperm cell stages outlined by Szmant-Froelich et al.^13^. In these samples, spermatocysts containing sperm in the final stage of spermatogenesis (spermatozoa) were plentiful and recognizable at lower magnifications (100–200x) by the presence and arrangement of sperm tails within the spermatocyst (Fig. 2a,b). At higher magnifications (≥ 400x), late-stage sperm could be further divided into a traditional Stage IV with a round nucleus, and an even later stage that we termed “Stage IVb”, in which the nucleus was further compressed into a cone shape and the spermatozoon took on an overall morphology similar to other anthozoan sperm^14,15^ (Fig. 2e). These observations were supported by our nuclear diameter measurements representing the full spectrum of sperm development in this species (Fig. 3a).

### Deep-fjord sites

#### Glacier Bay National Park and Preserve: Gloomy Knob and Central Channel

Samples from deep-fjord sites in GBNPP also contained all four stages of spermatocysts (Fig. 3b). In contrast to the plentiful late-stage sperm in GOA samples, only a small proportion of spermatocysts from the these sites contained late-stage sperm (3 of 158 spermatocysts examined). This may have been a result of the timing of sample collection (March), as the spawning season in this population is unknown and collection may have occurred after most of the mature spermatocysts had already been spawned, but the presence of a few late-stage sperm confirmed that the deep-fjord samples were capable of producing fully developed male gametes. Many of the spermatocysts not containing late-stage sperm contained sperm in very early stages of development instead, suggesting that our collection took place as one spawning season was finishing and another cohort of sperm was beginning to develop.

### Shallow sites

#### Holkham Bay: Tracy Arm and Endicott Arm

Only early and intermediate-stage spermatocytes, as indicated by larger nuclear diameters, were present in samples from the two shallow-fjord sites in Tracy and Endicott Arms, HB (Figs. 2c,d and 3c). The morphological structure of samples previously collected over a 16-month period from Tracy Arm^11^ was also investigated using transmission electron microscopy (Fig. 2d, Supplemental Information Table S2). No ultrastructural evidence of mature spermatozoa or late-stage spermatogenesis such as chromatin condensation or flagellar development was found in any sample despite their distribution across a full reproductive cycle^11^.

### Temperature

**Figure 4:**
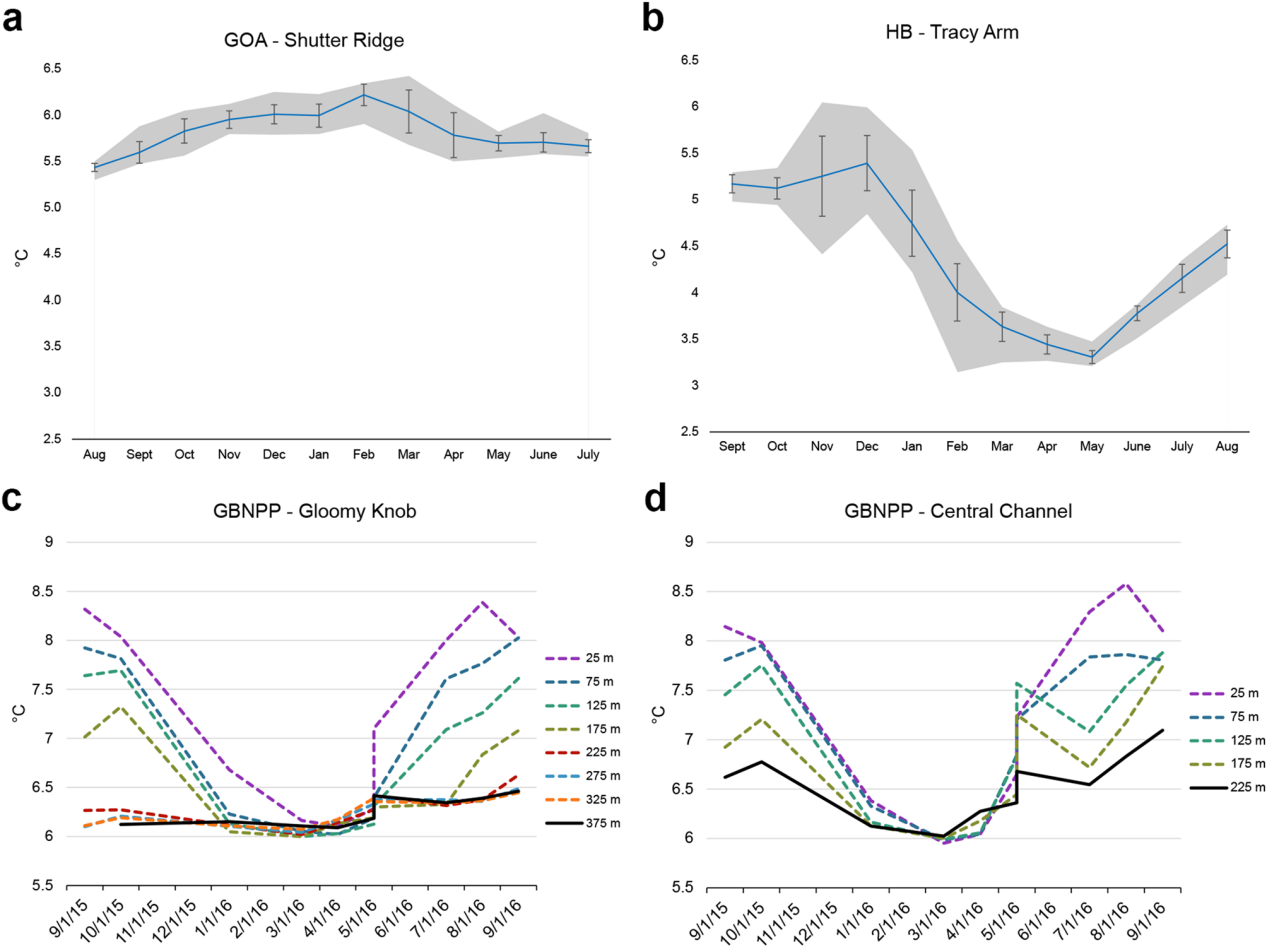
Annual temperature variability at GOA, HB, and GBNPP collection sites. (**a**,**b**) Temperature range and average monthly temperatures at deep-ocean (Shutter Ridge, **a**: 197 m) and shallow-fjord (Tracy Arm, **b**: 15 m) sites. (**c**,**d**) Temperature across depth at two deep-fjord sites (Gloomy Knob, **c**, and Central Channel, **d**) from monthly CTD casts in GBNPP. In (**c**,**d**), the solid black line indicates a depth similar to sample collection at that site.

At the time of collection, the mean monthly temperature at Shutter Ridge, a deep-ocean collection site, was 5.44 °C (SD = 0.04 °C), and the annual mean temperature was 5.84 °C (SD = 0.24 °C; Fig. 4a). At Central Channel, a deep-fjord collection site, the temperature measured at the time of collection was 6.08 °C, and, according to data from the National Park Service Southeast Alaska Inventory and Monitoring Network, the mean annual temperature was 6.55 °C (SD = 0.26 °C; Fig. 4d). At Tracy Arm, a shallow fjord collection site, the mean temperature during the months of collection were 5.17 °C (SD = 0.10 °C; September) and 3.63 °C (SD = 0.16 °C; March), and the annual mean temperature was 4.37 °C (SD = 0.75 °C; Fig. 4b).

Using the National Park Service Southeast Alaska Inventory and Monitoring Network’s long term oceanography cast data, we compared temperature trends across depth at Gloomy Knob and Central Channel in GBNPP based on monthly CTD casts and found that during the year of collection, thermal stability increased with depth (Fig. 4c,d). Further, over the last 20 years at the Central Channel collection site, the temperature of water below the thermocline at 245 m, has varied annually by 0.55–1.94 °C, (from an average low temperature of 4.60 °C to an average high temperature of 5.78 °C) while the temperature at 15 m (a similar depth to the shallow-fjord site in Tracy Arm) has varied by 2.75–4.54 °C (from an average low temperature of 4.32 °C to an average high temperature of 7.99 °C)^16^ (Supplemental Information Fig. S1). Thus, at the Central Channel collection site, the annual range of temperatures in shallow water has been more than twice that of the deep water below every year since 1999.

While full water column profile data are not available for the deep-ocean and shallow-fjord sampling locations, long-term temperature loggers deployed at these sites measured annual temperature ranges at Shutter Ridge and Tracy Arm that fit the pattern of greater variability in shallow waters (Fig. 4a,b). Temperatures at Shutter Ridge (208 m) varied by 1.12 °C during the year of collection (Fig. 4a), from a low temperature of 5.30 °C to a high temperature of 6.42 °C. Temperatures at Tracy Arm ranged by 2.91 °C during the year of collection, from a low temperature of 3.14 °C to a high temperature of 6.05 °C (Fig. 4b).

## Discussion

In this study we compared RTC spermatogenesis between shallow- and deep-water populations in Southeastern Alaska (above and below the seasonal thermocline), and though we found mature spermatozoa in individuals from the deep sites, spermatogenesis did not progress beyond an intermediate stage in individuals from the shallow sites. Of principal concern was the absence of structures critical for sperm function, such as flagella, in any shallow-water samples analyzed via histological or electron microscopy preparations. Additionally, no evidence was found in shallow colonies of sperm heads changing shape to the elongate triangular forms characteristic of mature cnidarian sperm^15^. Thus, we report that male gametogenesis is prematurely arrested in shallow RTC colonies. This finding, in conjunction with that of smaller oocyte sizes described previously in shallow RTC colonies^11^, leads us to suggest that the shallow, deep-sea emergent RTC populations may not be reproductively competent, may not produce viable larvae on regular timescales, and may be incapable of contributing to the local gene pool.

The most striking environmental difference in the shallow habitats compared with the deep sites is temperature, particularly thermal variability. Tide-water glaciers drive the oceanographic conditions that promote deep-sea emergence of RTC in shallow-water fjord habitats^10^. However, climate change has contributed to the rapid retreat and recent grounding of many glaciers, resulting in oceanographic changes that have put deep-sea emergent populations of RTC in conditions near the edge of their physiological tolerance^10^. In shallow-fjord habitats, temperatures can vary 2–7 times more than in deep-fjord habitats, and seasonally reach 7.5–8.0 °C (Fig. 4c,d, Supplemental Information Fig. S1), an apparently lethal condition for laboratory-held corals if sustained for 5 days or more (R. Stone, unpub. data). We suggest that more frequent and extreme temperature fluctuations^10,17,18^ may have caused physiological stress in shallow RTC populations that resulted in reproductive dysfunction.

The presence of shallow-water corals in the fjords suggests that, at least within the last few decades, conditions were suitable for recruitment there, perhaps from populations below the thermocline where temperatures are more stable. We have, however, no evidence to determine whether gametogenic arrest in shallow-water populations is a recent condition or a longstanding one. Adverse conditions aside from variable temperatures in the fjord environment, including high turbidity and silt load^10^, may have affected or prevented reproduction from occurring. Alternatively, the shallow-water populations may have once achieved successful reproduction and only recently been affected by these stressors to the point of gametogenic arrest. The RTC colonies from GBNPP (deep-fjord sites) generally had fewer spermatocysts than those from other sites, such that more colonies were examined to analyze a comparable number of spermatocysts to the other study sites. We attributed fewer present spermatocysts in each colony to the timing of collection in relation to spawning, however we cannot exclude the possibility that oceanographic factors contributed to the lack of spermatocysts in RTC from the deep-fjord sites in GBNPP.

Although we have focused primarily on high-temperature stress as a cause of reproductive disruption, the shallow-fjord RTC habitats also experienced cooler temperatures than those at our deep-water sites. *P. pacifica* populations occur throughout the northern North Pacific Ocean, and the family Primnoidae is well documented in cold-water regions worldwide, including the Bering Sea and Antarctic waters^19,20^. But as we report here, the presence of apparently healthy colonies may or may not indicate reproductive health. For example, apparently self-sustaining RTC populations have been documented at temperatures as low as 0.2 °C on an isolated seamount in the Sea of Japan^2^. Reproductive health of corals in that population was not examined, however their spatial separation from any source of recruits, unlike the shallow-fjord population in this study, suggests that recruitment (and hence successful reproduction) was from within the population persisting in colder conditions than anywhere in Alaska. Still, we cannot conclusively rule out low temperature stress as a contributing factor in reproductive dysfunction and urge that future research priorities include studies on low-temperature tolerance with regard to gametogenesis and studies of larval dynamics and recruitment.

Our results indicate that thermal stability within an optimum range is a key factor for successful spermatogenesis in RTC. We suggest that healthy reproduction at deep sites below the thermocline is supported by stable temperatures, which, although seasonally warmer than shallow sites, remain below the observed lethal range for *P. pacifica.* If, as we suggest, thermal variation is a causative agent of reproductive dysfunction, predictions for the GOA under various climate change scenarios are concerning for deep-ocean RTC populations there. In addition to substantial warming predicted for the GOA, warm water events in the region are expected to become more frequent^17,18^. Temperature loggers moored on the seafloor at deep-ocean sites in the eastern GOA between August 2013 and June 2015 provided valuable insights into the thermal regime presently experienced by RTCs. In 2014, the GOA began to experience a marine heatwave that reportedly affected our Shutter Ridge study site^18,21^ (Supplemental Information Table S3). Temperatures at another site in the GOA with documented RTC populations reached as high as 10 °C (R. Stone unpub. data). We collected and analyzed our samples from Shutter Ridge in 2013 and confirm that colonies at that time were reproductively healthy; it remains to be seen if the subsequent warm-water event affected RTC reproduction thereafter.

Environmental stress affects reproductive potential in corals^22^ as limited energetic reserves are partitioned between basic core processes (homeostasis, reproduction, growth) and responding to stressors^23^. The physiological consequences of thermal stress on adult cold-water corals vary among species, but generally, warmer temperatures have been linked to increased mortality^24,25^, decreased calcification rates^26^, and delayed recovery from short-term pollution exposure^27^. In some warm-water corals, exposure to increased thermal variability has been shown to promote acclimatization^28–30^, potentially driving directional selection for more heat-tolerant genotypes over time^28^. However, these studies have largely focused on shallow zooxanthellate coral species and their responses to bleaching events, and few have explicitly considered the impacts of repeated thermal stress or thermal variability on reproduction (but see Liberman et al*.*^31^). Results of warming experiments^26,27^ and descriptions of reproduction and metabolic demand across environmental gradients^32–36^ suggest that the energetic requirements of survival in suboptimal temperatures, particularly during gametogenesis when metabolic demand is already elevated^35^, may not be sustainable across coral species^33,36–38^. Living in suboptimal environments has also been shown to affect reproductive output in corals^32,36^. In some cases, this appears as a temporal offset across depths, possibly due to differences in temperature between habitats^34,35,39–41^. In other species, changes in reproductive phenology can disrupt broadcast spawning synchrony^42^. In others still, less healthy coral individuals experiencing greater physiological stress than their conspecifics produce fewer offspring, reducing their reproductive output as they manage homeostasis^38^. A common theme among these studies is that the native, unaltered spatial range of a given coral species is often the ideal environment for reproduction, and while deviations from those conditions may not be lethal to the parental stock, they can produce similar sub-lethal reproductive effects to those we report for RTC here. In other marine invertebrates, increased temperature has been shown to disrupt reproduction by negatively impacting gametogenesis (echinoderms^43^), reducing gamete quality (echinoderms^44^), and reducing spawning and spermatogenesis (bivalves^45^; cephalopods^46^).

While the presence of non-reproductive adult colonies is an obvious indication of physiological stress, the presence of reproductively incompetent colonies is much less outwardly apparent. The description of reproductive dysfunction as a cryptic intermediate thermal stress response highlights the important distinction between species presence and reproductive capacity^36,38,42^. Our findings demonstrate that the absence of terminal (and, as we suggest, functional) gametogenic stages may be a subtle, but impactful, consequence of life near the physiological limit. Unlike immature or wholly non-reproductive colonies characterized by a complete lack of gametes, these affected colonies produce gametes that progress through some of the gametogenic program but are not fully formed and lack key functional features, likely reducing or inhibiting the production of larvae.

An important aspect of this dysfunction unique to deep-sea organisms is how easily it might be overlooked in standard deep-sea collections. The deep-sea environment is remote, requiring resource intensive, logistically complex sampling efforts. In many cases, sampling is inadequate and rarely can the same individual or population be resampled, an important aspect of any scientific study. Often, descriptions of reproductive seasonality in deep-sea organisms occur at the same time as the initial description of gametes in that species, resulting in a limited data set and a lack of temporal perspective. In the absence of the context provided by examples of late-stage gametes and a repeated, comprehensive sampling protocol, both of which are often lacking in deep-sea species due to sampling constraints, reproductive dysfunction as observed here in RTC would undoubtedly have gone undetected. In Tracy Arm, males progressed through a seasonal pattern of spermatogenesis, but when compared with a late-stage spermatozoan from a deep-water site, it became clear that, despite the seasonal pattern, sperm were not maturing completely. This example highlights the importance of late-stage gametes as context to identify reproductive dysfunction. Comprehensive sampling is also key, as the lack of mature sperm in a single sample would be disregarded as a feature of reproductive seasonality rather than flagged as evidence of reproductive dysfunction. The findings reported here were only possible because the Tracy Arm colonies were studied extensively over 16 months, in comparison to the other sites, which were sampled once or twice. The lack of mature sperm in Tracy Arm despite this thorough sampling emphasizes the difference in the condition of gametes between colonies from Tracy Arm and the deep water sites, which contained late-stage sperm despite being sampled so much less.

The protection of several major RTC thickets in the eastern GOA as Habitats of Particular Concern in 2006^1,9^ was an important first step in the conservation of fisheries habitat. However, one of the underlying principles of the closures was to provide sanctuary to large concentrations of reproductive colonies that were a source of recruits for unprotected coral habitats in the region. Our findings challenge that principle, suggesting that protected RTC thickets might not be reproductively healthy in a changing GOA. In the coming decades, deep-ocean coral habitats in the GOA that currently host thriving *P. pacifica* populations are expected to experience temperature regimes similar to those presently occurring in the fjord environments ^17,21,47^ and will likely experience additional extreme events such as the marine heatwave of 2014–2016^21^. These thermal predictions, if realized, threaten the reproductive health of the corals forming massive thickets at depth, suggesting that, like their shallow-water fjord counterparts, they may soon be bathed in seawater at the limit of their physiological tolerance. Given the importance of RTCs as fisheries habitat in the Northeast Pacific Ocean we strongly urge that additional studies on their reproductive ecology (including thermal tolerance) and larval dispersal be undertaken and that the coral thickets in the eastern GOA be highlighted for state-of-the-art oceanographic monitoring now.

## Methods

All *Primnoa pacifica* samples for this study (Supplemental Information Tables S1, S2) were collected with SCUBA or Remotely Operated Vehicles (ROV)^4,10^. Histological samples were initially prepared and examined using the techniques described by Waller et al*.*^4,11^.

### Deep-ocean sites—Gulf of Alaska: Shutter Ridge and Dixon Entrance

Shutter Ridge and Dixon Entrance are located on the continental shelf in the eastern Gulf of Alaska (GOA) on the continental shelf edge. Shutter Ridge is a series of pinnacles located 28 km west of Cape Ommaney, Baranof Island^1^. Dixon Entrance is a deep basin that separates northern British Columbia (Canada) and Southeast Alaska. It is partially bounded by glacially scoured bedrock and littered with glacial dropstones^48^. Shutter Ridge colonies were sampled using the ROV *H2000* (Deep Ocean Exploration and Research) in August 2013 at depths between 191 and 196 m. Dixon Entrance colonies were sampled with the ROV *Zeus II* (Pelagic Research Services) in June 2015 at depths between 165 and 347 m. Three male specimens were selected for analysis from the Shutter Ridge collection and five from the Dixon Entrance collection (Supplemental Information Table S1).

### Deep-fjord sites—Glacier Bay National Park and Preserve: Gloomy Knob and Central Channel

Glacier Bay is located in the northern inside waters of Southeast Alaska and is a system of glacially derived fjords with two main fjords (the East and West Arms) branching from the Central Channel^10,49^. The bay is strongly influenced by tidewater glaciers, including McBride Glacier in the East Arm, and Johns Hopkins, Margerie, and Lamplugh Glaciers, in the West Arm^10^. The two arms of the fjord join in the main section of the bay known as the Central Channel and the main bay continues out over the terminal moraine through Icy Strait which flows into the eastern GOA^49^. Gloomy Knob is located in the West Arm, 40 km from the head of the fjord^49^. The Central Channel is an area of relatively flat seafloor and is influenced by glacial water from both arms. Colonies at both sites were sampled with the ROV *Kraken II* (University of Connecticut) in March 2016 at depths between 385–392 m (Gloomy Knob, N = 13) and 242–295 (Central Channel, N = 30). From those collections, 8 and 10 male specimens were selected for analysis, respectively (Supplemental Information Table S1).

### Shallow-fjord sites—Holkham Bay: Tracy and Endicott Arms

Tracy and Endicott Arms are the northern and southern arms of Holkham Bay^10^. Tracy Arm has a maximum depth of 378 m and terminates in two rapidly retreating tidewater glaciers, the Sawyer and South Sawyer^10^. The Tracy Arm collection site is located 14.5 km from the head of the fjord on a vertical wall composed of graywacke and graniodorite^4,10,11^. Tagged colonies (N = 38) were sampled with SCUBA every 3 months over a 16-month period (September 2010-January 2012), at depths of 10–17 m^11^. Additionally, some of these colonies were resampled in January 2013 and February 2018. From the data compiled from all these collections, five male specimens, including three representing the latest stage of spermatogenesis present in any sample from the Waller et al*.* 2014 study were selected for analysis (Supplemental Information Tables S1, S2). These colonies were all collected from depths of 11–12 m (Supplemental Information Table S1).

Endicott Arm reaches more than 350 m deep and terminates at the tidewater Dawes Glacier^4^. The collection site, where colonies were sampled by SCUBA and ROV in June 2014 at depths between 16–23 m, is 7.9 km from the head of the fjord^4,10^. From that collection three male specimens were selected for analysis (Supplemental Information Table S1).

### Histology

All specimens used in this study had been previously assayed using histological techniques to describe reproductive seasonality: colonies had been sexed, oocytes had been measured and spermatocysts had been staged (GOA, HB: see Waller et al.^4,11^; GBNPP: Waller et al*.*^4^, Waller unpub. data). All specimens that had been previously prepared for Waller et al*.* 2014^4^ and Waller et al*.* 2019^11^ were reexamined and 3–10 samples from each site were identified that would provide a full complement of spermatocyst stages, especially the latest stages, according to the sperm staging protocol previously developed for the species^11^ and adopted by researchers thereafter^4,50^. The aim was to measure nuclei from 30 spermatocysts of each stage present, which in some populations required only a few individuals, but in other populations with fewer spermatocysts per colony, required more individuals be included to meet that target. The presence of multiple spermatocyst stages in each sample and overlap in spermatogenic cohorts combined with the natural variation between samples provided coverage over the complete range of spermatogenic stages without the collection of a time series. These samples were re-assayed at higher magnification (600 × and 1000x) under oil immersion using an Olympus BH-2 compound microscope to identify characteristics that would indicate late-stage spermatozoa such as the presence of tails and narrowed, conical sperm nuclei^13^. Due to meiotic divisions during spermatogenesis, the nuclear diameter of individual spermatocytes decreased as spermatogenesis progressed^12^. Images of spermatocysts were captured using an Olympus BH2 compound microscope fitted with an InfinityPro1 camera and InfinityCapture software. The feret diameter of individual spermatocyte nuclei were measured using ImageJ.

### Electron microscopy

In addition to the histological analysis, select coral tissue from each of the eight sampling periods (September 2010-January 2013) in Tracy Arm^11^ were preserved for electron microscopy. In specimens collected between September 2010 and January 2013, the samples were preserved immediately upon collection and later shipped to the Darling Marine Center for analysis. Of these, 26 specimens were examined for ultrastructural indications of mature spermatozoa (Supplemental Information Table S2).

Samples for electron microscopy were preserved by immediately excising polyps containing gametes (oocytes and spermatocysts) and fixing them in 4% glutaraldehyde in 0.1 M sodium cacodylate buffer for 1 h, washing them in 0.1 M sodium cacodylate with 0.4 M sucrose for 1 h, then post-fixed with 0.1% osmium tetroxide in 1 M sodium cacodylate for 40–60 min. Samples were kept at 4 °C throughout the fixation process then dehydrated to 70% ethanol.

Full polyps and spermatocysts sampled for transmission electron microscopy were further dehydrated in a graded ethanol series, washed in propylene oxide (three washes for 10 min each), and embedded in Epon (EMBed-812 Embedding Kit, Electron Microscopy Sciences). Semi-thin (0.5 micron) and ultrathin sections were cut on a Porter-Blum MT2-B ultramicrotome. Semi-thin sections were mounted on glass slides, stained with Richardson’s stain, and cover-slipped. These sections were examined and imaged at 600 × under oil immersion using an Olympus CX31 compound microscope. Ultrathin sections were mounted on G-200 copper grids and stained with aqueous uranyl acetate and lead citrate. Ultrathin sections were examined and imaged using a Philips CM10 transmission electron microscope operated at 100 kV and fitted with a Gatan Orius 830 camera running Digital Micrograph.

### Temperatures

Temperatures were collected every six hours from temperature loggers (DST CTD, Star Oddi and Minilog 12-TR, VEMCO) deployed at the study sites at Shutter Ridge from August 2013 to June 2015 (GOA, R. Stone unpub. data) and Tracy Arm (HB)^11^ from September 2010 to January 2012. Monthly temperatures from these sites were generated by averaging the temperatures collected over the month.

GBNPP long-term (1999-present) temperature data were collected during monthly oceanographic surveys at established sites by the National Park Service Southeast Alaska Inventory and Monitoring Network^16^. At our study sites, Gloomy Knob and Central Channel, CTD (conductivity, temperature, and depth) profiles were collected to depths of over 400 and 300 m, respectively.

## Supplementary Information

Supplementary Information.

## Data Availability

The data that support the findings of this study are available from the corresponding author upon reasonable request.
