# Supplementary material for: Shallow-emerged coral may warn of deep-sea coral response to thermal stress
Source: Sci Rep. 2021 Nov 17;11:22439. doi: 10.1038/s41598-021-01948-2 (PMC8599838; doi:10.1038/s41598-021-01948-2)
Supplement: Supplementary file 1 — Supplementary Information. [file 41598_2021_1948_MOESM1_ESM.docx]

**Supplementary Information:**

**Title: Shallow-emerged coral may warn of deep-sea coral response to thermal stress**

**Authors:** Julia W. Johnstone^1*^, Rhian G. Waller^1^, Robert P. Stone^2^,

Supplemental Information Table S1. Collection information, colony measurements, and gamete data for the specimens histologically examined in this study. Detailed collection information for the Tracy Arm (*) samples can be found in Supplemental Information Table S2.

| **Region** | **Site** | **Date Sampled** | **Number of Colonies (N)** | **Depth range (m)** | | **Colony Height (cm)** | | **Spermato-cysts Analyzed (N)** | **Nuclei Measured (N)** | **Mean nuclear diameter (μm)** | |
| --- | --- | --- | --- | --- | --- | --- | --- | --- | --- | --- | --- |
|  |  | **Month/**  **Year** |  | **Min.** | **Max.** | **Min.** | **Max.** |  |  | **Min.** | **Max.** |
| GOA | Dixon Entrance | June 2015 | 5 | 165 | 347 | 30 | 130 | 120 | 9245 | 1.09 | 5.36 |
| GOA | Shutter Ridge | August 2013 | 3 | 191 | 196 | 65 | 180 | 120 | 9145 | 1.1 | 4.66 |
| GBNPP | Gloomy Knob | March 2016 | 8 | 385 | 392 | 109 | 155 | 66 | 3873 | 1.27 | 5.21 |
| GBNPP | Central Channel | March 2016 | 10 | 242 | 295 | 29 | 104 | 92 | 5190 | 1.48 | 5.38 |
| HB* | Tracy Arm | Sept. 2010  March 2011 | 5 | 11 | 12 | 95 | 151 | 90 | 6185 | 2.83 | 5.14 |
| HB | Endicott Arm | June 2014 | 3 | 16 | 23 | 90 | 100 | 67 | 4265 | 2.5 | 4.93 |

Supplemental Information Table S2. Collection information and raw spermatocyst staging data for *P. pacifica* from Tracy Arm for TEM and nuclear size analysis in this study. Spermatocyst staging data was originally published in Waller *et al*.^11^. These samples were selected for TEM analysis because they represented the most productive males from the 2014 study. Shaded samples were also used for sperm nuclear sizing to provide a representation of the full complement of spermatogenesis, starting with early stages through the latest stages present in any sample. Results of the nuclear size analysis are presented in Figure 3 and Supplemental Information Table S1.

| **Original Sample ID** | **Month Collected** | **% spermatocysts determined to be late stage (3 or 4) by Waller *et al.* 2014** | **% spermatocysts determined to be stage 4 by Waller *et al.* 2014** | **# late stage spermatocytes found by EM** |
| --- | --- | --- | --- | --- |
| 67 | 9/10 | 52.4 | 16.2 | 0 |
| 71 | 9/11 | 61.3 | 15.5 | 0 |
| 85 | 3/11 | 40 | 12.8 | 0 |
| 94 | 9/10 | 63.7 | 25.4 | 0 |
| 321 | 9/10 | 53.5 | 29 | 0 |
|  | 1/13 | 9.2 | 2 | 0 |
| 349 | 9/10 | 53.2 | 1.3 | 0 |
|  | 3/11 | 17.4 | 0.9 | 0 |
|  | 9/11 | 45 | 1.3 | 0 |
|  | 1/13 | 6.4 | 0 | 0 |
| 350 | 9/10 | 45.7 | 0.7 | 0 |
|  | 9/11 | 0 | 0 | 0 |
|  | 1/13 | 0 | 0 | 0 |
| 361 | 9/10 | 0 | 0 | 0 |
|  | 9/11 | 64 | 20.7 | 0 |
|  | 1/13 | 3 | 0 | 0 |
| 362 | 3/11 | 3.4 | 0 | 0 |
|  | 9/11 | 64 | 20.7 | 0 |
| 411 | 9/10 | 53.5 | 28.3 | 0 |
|  | 3/11 | 47.4 | 9.9 | 0 |
|  | 9/11 | 44.2 | 5.8 | 0 |
|  | 1/13 | 17.6 | 1.6 | 0 |
| 494 | 9/10 | 72.5 | 32.4 | 0 |

|  | **Shutter Ridge**  **197 m MLLW** | | |
| --- | --- | --- | --- |
|  | **Max** | **Average** | **St.Dev** |
| **Aug 2013** | 5.50 | 5.44 | 0.04 |
| **Sept 2013** | 5.88 | 5.60 | 0.12 |
| **Oct 2013** | 6.05 | 5.83 | 0.13 |
| **Nov 2013** | 6.12 | 5.95 | 0.09 |
| **Dec 2013** | 6.25 | 6.01 | 0.10 |
| **Jan 2014** | 6.23 | 6.00 | 0.13 |
| **Feb 2014** | 6.34 | 6.22 | 0.12 |
| **Mar 2014** | 6.42 | 6.04 | 0.23 |
| **Apr 2014** | 6.11 | 5.78 | 0.24 |
| **May 2014** | 5.82 | 5.70 | 0.08 |
| **Jun 2014** | 6.02 | 5.71 | 0.11 |
| **Jul 2014** | 5.81 | 5.66 | 0.07 |
| **Aug 2014** | 5.88 | 5.73 | 0.07 |
| **Sept 2014** | 5.90 | 5.72 | 0.07 |
| **Oct 2014** | 6.47 | 6.05 | 0.20 |
| **Nov 2014** | 7.38 | 6.65 | 0.27 |
| **Dec 2014** | **7.65** | 6.86 | 0.25 |
| **Jan 2015** | 7.03 | 6.70 | 0.16 |
| **Feb 2015** | 7.09 | 6.68 | 0.19 |
| **Mar 2015** | 6.53 | 6.25 | 0.13 |
| **Apr 2015** | 6.13 | 5.97 | 0.08 |
| **May 2015** | 5.94 | 5.72 | 0.12 |

Supplemental Information Table S3. Monthly temperatures at Shutter Ridge from August 2013 to May 2015. Temperatures over 7.5°C are shown in bold and shaded.

|  |
| --- |
| Supplemental Information Figure S1. Multi-year patterns in thermal variability with depth at Central Channel in GBNPP. Using the National Park Service Southeast Alaska Inventory and Monitoring Network monthly CTD cast data set, this plot was assembled to compare annual temperature variability at different depths. The blue bars show the annual range of temperatures at approximately the depth of our collection, 245 m, while the grey bars show the annual range of temperatures at that site at a depth similar to our shallow fjord site in HB, 15 m. |
